# Oral cholera vaccine coverage in hard-to-reach fishermen communities after two mass Campaigns, Malawi, 2016

**DOI:** 10.1016/j.vaccine.2017.07.104

**Published:** 2017-09-12

**Authors:** Delphine Sauvageot, Christel Saussier, Abebe Gobeze, Sikhona Chipeta, Innocent Mhango, Gift Kawalazira, Martin A. Mengel, Dominique Legros, Philippe Cavailler, Maurice M'bang'ombe

**Affiliations:** a Agence de Médecine Préventive, Paris, France; b UNICEF, Malawi Country Office, Lilongwe, Malawi; c Ministry of Health, Community Health Sciences Unit, Lilongwe, Malawi; d Ministry of Health, District Health Office, Machinga, Malawi; e Ministry of Health, District Health Office, Zomba, Malawi; f World Health Organization, Geneva, Switzerland

**Keywords:** AEFI, Adverse Event Following Immunization, AMP, Agence de Médecine Préventive, DOV, Directly Observed Vaccination, HH, Household, MoH, Ministry of Health, MSF, Médecins Sans Frontières, OCV, Oral Cholera Vaccine, VC, Vaccine Coverage, WASH, Water Sanitation Hygiene, Cholera, Oral cholera vaccine, Fishermen, Vaccine coverage, AEFI, Malawi

## Abstract

**Context:**

From December 2015 to August 2016, a large epidemic of cholera affected the fishermen of Lake Chilwa in Malawi. A first reactive Oral Cholera Vaccines (OCV) campaign was organized, in February, in a 2 km radius of the lake followed by a preemptive one, conducted in November, in a 25 km radius. We present the vaccine coverage reached in hard-to-reach population using simplified delivery strategies.

**Method:**

We conducted two-stage random-sampling cross-sectional surveys among individuals living in a 2 km and 25 km radius of Lake Chilwa (islands and floating homes included). Individuals aged 12 months and older from Machinga and Zomba districts were sampled: 43 clusters of 14 households were surveyed. Simplified strategies were used for those living in islands and floating homes: self- delivery and community-supervised delivery of the second dose. Vaccine coverage (VC) for at-least-two-doses was estimated taking into account sampling weights and design effects.

**Results:**

A total of 1176 households were surveyed (2.7% of non-response). Among the 2833 individuals living in the 2 km radius of Lake and the 2915 in the 25 km radius: 457 (16.1%) and 239 (8.2%) lived in floating homes or on islands at some point in the year, respectively. For the overall population, VC was 75.6% and 54.2%, respectively. In the 2 km radius, VC was 92.2% for those living on the lake at some point of the year: 271 (64.8%) used the simplified strategies. The main reasons for non-vaccination were absence during the campaign and vaccine shortage. Few adverse events occurring in the 24 h following vaccination was reported.

**Conclusions:**

We reached a high two-dose coverage of the most at-risk population using simplified delivery strategies. Because of the high fishermen mobility, regular catch-up campaigns or another strategy specifically targeting fishermen need to be assessed for more efficient vaccines use.

## Highlights

• Simplified delivery strategies were proposed in hard-to-reach population.
• High full coverage was reached in the most at-risk individuals.
• Vaccines use efficiency need to be improved.

## 1 Introduction

Despite improvements in access to safe water provision and proper sanitation, cholera remains a significant public health concern in Malawi. The Lake Chilwa located in the Southern of Malawi has a turbidity and a mineral content ideal for Vibrio cholerae. The population living in the three districts surrounding the lake (Machinga, Zomba and Phalombe), mainly fishermen and their families, were identified as having a high-risk for cholera [1]. Some fishermen, from 3000 in low fishing season to approximately 10,000 in high season, leave their home village for living in the islands or in the *Zimboweras* (temporary floating homes) by groups of 10–16 individuals. They stay for from one up to three months without coming ashore and use the lake for drinking, defecating, bathing, and cooking. As it takes around three hours by canoe to reach the land, they have a poor access to health care [2], [3].

Cholera cases were reported annually in this area with large outbreaks occurring in 2002, 2010, 2012, and 2016. Between January and September 2016, a total of 1256 suspect cholera cases were notified mainly affecting fishermen [2], [3]. In response to this epidemic, a two-dose mass campaign with oral cholera vaccine (OCV) using Shanchol (Shanta-Biotechnics, Hyderabad, India) was conducted in February-March 2016 by the Ministry of Health (MoH), with the support of *Médecins Sans Frontières* (MSF)-France, and *Agence de Médecine Préventive* (AMP). A total of 190,000 vaccine doses were delivered to individuals living within a 2 km radius of the lake, on the shore, the islands and *Zimboweras* located in the three districts surrounding the lake. In March-April 2016, the two-dose vaccine coverage was 91% in islands, 79% in *Zimboweras* and 53% on the shore [4].

However, additional fishermen moved to the lake for fishing and cholera cases continued to be notified until September 2016 in the Machinga and Zomba districts. Because of frequent population movement from the villages in land to the lake and a cholera outbreak difficult to control, the MoH decided to conduct a second OCV campaign at the end of the fishing season in order to vaccinate the remaining seasonal fishermen and other at-risk individuals in their home village. In November 2016, 180,000 doses of Euvichol vaccines (EuBiologics, Seoul, South Korea) were delivered, targeting all individuals aged 12 months and older, not fully vaccinated, living within 25 km radius of the lake in Machinga and Zomba districts.

This paper summarizes the results of the cholera immunization coverage survey conducted in December 2016 by the MoH with the support of UNICEF and AMP, in populations living in a 2 km radius of the lake (the most at-risk population because of their proximity with the lake) and in the 25 km radius of the lake (considered as part of the cholera hotspot by the MoH). The primary objective of the survey was to assess the vaccine coverage reached after the two successive OCV campaigns, in the both targeted areas. The secondary objectives were to describe the vaccine coverage per age groups and in specific at-risk individuals such as people living in islands or *Zimboweras* at some point of the year. The reasons of non or partial vaccination, the mode of vaccine dose delivery, the vaccine side effects following Euvichol oral vaccine administration were detailed.

## 2 Methods

### 2.1 Presentation of the OCV campaigns

The first OCV campaign took place between February 16 and 22, 2016 (1st round) and the second round between March 9 and 15, 2016 (2nd round). The targeted population was living in a 2 km radius of the lake, including the islands and fishermen settled in *Zimboweras*, in the districts of Machinga, Zomba, and Phalombe. All individuals received their first dose via the standard method, Directly Observed Vaccination (DOV) at immunization points. For the second dose, two innovative simplified delivery strategies were used. During the first round, those living in *Zimboweras* were given an extra dose on the same day of the first dose intake and were instructed to drink it at home 14 days after the first dose (self-delivery strategy). On the islands, at the end of the first round, a stock of vaccines was entrusted to the community leaders who distributed them, fourteen days after the first round, to the households heads for home-based self-administration (community supervised delivery strategy).

The second OCV campaign was conducted nine months after the first one. The target population was living in a 25 km radius, including the islands and the *Zimboweras* in Machinga and Zomba districts*,* two out of the three districts surrounding the lake. The first round was carried out between November 14 and 20, 2016 and the second between December 5 and 9, 2016. The first and the second dose were delivered by DOV at 110 immunization points. In both *Zimboweras* and islands, only the self-delivery strategy for the second dose was used.

The immunization cards were systematically distributed during each campaign. At immunization point, the vaccinators recorded on the cards the date of the dose administration and the scheduled date for the next one. If one individual was two-dose vaccinated in February-March, he was not vaccinated in November-December. If the self-delivery strategy was used, only the scheduled date for the 2nd dose administration was recorded. If the community-supervised delivery strategy was used, the community leader recorded the date of second dose delivery on the card.

### 2.2 Cross-Sectional surveys

All individuals’ aged 12 months and older (target age group for OCV), residents of Machinga and Zomba districts, within a 2 km radius of lake (target population for the first campaign) or a 25 km radius (target population for the second campaign), were eligible for inclusion in the coverage surveys. All the islands located in Machinga and Zomba districts (Chisi, Chidyamphiri, Thongwe, and Chinguma) were included in the targeted areas. Two representative samples of the population were selected using a cluster-based two-stage random sampling: one in the 2 km radius of the lake and one in the 25 km radius of the lake. Given that the two target areas geographically overlap, populations living on the islands, the *Zimboweras*, and in the 2 km radius can be sampled for both surveys.

The sample size was calculated to obtain an estimate of the proportion of individuals who received two doses of OCV by age group (1–4, 5–14, 15 years and older) in each targeted area. Sample sizes were calculated to ensure a sufficiently precise estimate for children aged 1–4 years as this group was the smallest. We considered the following assumptions: 70% two doses vaccine coverage, a design effect (DE) of 3.0 (potential heterogeneity of clusters for immunization), 8% precision and 95% confidence interval (CI). According to the 2010 Demographic and Health Survey, the mean household (HH) size was four individuals with 18% of children 1–4 years old. Assuming 12% of absence or refusals, we, therefore, planned to survey 602 households (43 clusters of 14 HH) in each targeted area. A cluster was a group of households selected by proximity in one given village. A household was defined as a group of people sleeping under the same roof and sharing meals every day for at least the previous two weeks. Villages were randomly selected proportionate to their population size using the December 2016 census performed by the health community workers (221,051 inhabitants estimated within 25 km radius of the lake). The sampling frame was elaborated for the survey purpose. Once the villages were identified, a direction was selected from the geographical center of the village using the “spinning a pen on the ground” method. All the houses in this direction were counted then one (the first household of the cluster) was randomly selected using a random number table. The subsequent homes to be visited will be the second nearest one at right hand until the 14 households were enrolled. In each household, all individuals older than one year old were included.

A standardized, pre-piloted questionnaire was used. We collected data on age, sex, duration in months spent in islands or Zimboweras in the past fishing season, the mode of vaccine administration (DOV, self administration, community supervised delivery) and the place of dose delivery. To avoid potential memory bias, the mode of information and the adverse events in the 24 h following immunization were collected only for the campaign of November-December. The vaccination status was assessed first by recall. All the household members at home at the time of the survey were requested to be present. If one was absent, the female head of household responded on behalf of him. The vaccination status was also assessed based on immunization cards, when available. The dates of each vaccine dose administration were collected (on recall and based on immunization card). If no card or missing information, the date at mid-period of the vaccination round was collected. We developed a calendar of local events for helping with dates.

Vaccine coverage was therefore estimated using individuals’ recall and immunization card. Vaccine coverage par age group (1–4 years old, 5–14 years old, 15 year old and older) and for individuals living in islands or *Zimboweras* at some point of the year were estimated using individual recall only.

Interviews were conducted in the local language by 14 teams composed of two surveyors. The field work was monitored by four supervisors and two coordinators oversaw the survey. All surveyors and supervisors were recruited locally and participated in a two-day training session. Surveyors conducted face-to-face interviews after oral consent was given from the head of the household. If all the household members were absent, the team had to come back the same day. If the occupants were still absent during the second visit or refused to participate, that household was replaced.

### 2.3 Data entry and analysis

Data entry was performed using EpiData 3.1 (EpiData Association, Denmark) and data analysis was performed using Stata 12.0 (StataCorp, College Station, Tx, USA). Point estimates and 95% Confidence Interval (CI) were calculated considering the sampling weight and the design effect. Results were presented for the 2 km radius area and the 25 km radius area.

### 2.4 Ethical considerations

The Malawian National Health Sciences Research Committee approved the study protocol.

## 3 Results

Of the required 86 clusters randomly selected (43 for each survey), four were used for the two surveys, given that the two target areas naturally overlap. A total of 1176 households were visited: 1144 were included, 22 were absents and 10 refused to participate leading to a non-response proportion of 2.7%.

A total of 2833 individuals were surveyed in the 2 km radius of lake Chilwa and 2915 in the 25 km radius. Respectively, 16.1% (n = 457) and 8.2% (n = 239) reported living on the lake at some point of the year while 5.7% (n = 163) and 3.0% (n = 86) reported being fishermen (Table 1). For both target population, people reported being informed about the organization of the November-December campaign via megaphones in villages (60.6% and 47.2% for the 2 km and 25 km radius, respectively) and at health centers (28.5% and 36.6% for the 2 km and 25 km radius, respectively) (Table 2).

**Table 1:** Characteristics of population surveyed in a 2 km (N = 2833) and 25 km (N = 2915) radius of Lake Chilwa, Malawi, December 2016.

		Population in a 2 km radius N = 2833		Population in a 25 km radius lake N = 2915	
		n	%	n	%
Age group (years)					
	1–4	519	18.3	575	19.7
	5–14	901	31.8	968	33.2
	≥15	1412	49.8	1372	47.1
	Not reported	1	0.04	0	*NA*

Sex					
	Female	1425	50.3	1453	49.9
	Male	1398	49.4	1446	49.6
	Not reported	10	0.4	16	0.6

Residence at survey time					
	*Zimboweras*	80	2.8	0	*NA*
	Island	254	9.0	147	5.0
	Villages in 2 km radius of Lake	2499	88.2	1017	34.9
	Villages in 3–25 km radius of Lake	0	*NA*	1751	60.1

Occupation					
	Farmer	810	28.6	880	30.2
	Fisherman	161	5.7	84	2.9
	Fish preparation/seller	32	1.1	15	0.5
	Business	122	4.3	137	4.7
	Student	970	34.2	986	33.8
	Pre-school child (<6 yr)	618	21.8	680	23.3
	Other	113	4.0	131	4.5
	Not reported	7	0.3	2	0.1
Live in floating homes or on the islands at some point of the year					
	Yes	457	16.1	239	8.2

*NA*: Not applicable.

**Table 2:** First source of information on OCV campaign in population living in a 2 km (N = 2833) and 25 km (N = 2915) radius of Lake Chilwa, Malawi, December 2016.

	Population in a 2 km radius of the lake N = 2833		Population in a 25 km radius of the lake N = 2915	
	n	%	n	%
Megaphone	1718	60.6	1231	42.2
Health center before the campaign	808	28.5	1067	36.6
Relatives/friends	213	7.5	396	13.6
Radio	31	1.1	50	1.7
Other	35	1.3	67	2.3
Not reported	28	1.0	1	0.03

In the 2 km radius, 65.9% of individuals (n = 1697) received at least one dose of vaccine during the February-March campaign, 27.9% (n = 718) during the November-December campaign, and 5.9% (n = 153) during both campaigns (missing information, n = 7); in the 25 km radius (missing information, n = 8), the proportions were 30.2% (n = 706), 66.8% (n = 1558) and 2.6% (n = 60), respectively. In both campaigns combined, the second dose self-delivery strategy was proposed to 4.8% (n = 125) of individuals in the 2 km radius and to 0.6% (n = 14) in the 25 km radius (missing information, n = 13). The community supervised strategy was proposed to 5.8% (n = 149) and to 3.5% (n = 81), respectively.

After the two successive campaigns, the at-least one-dose self-reported OCV coverage, was 91.4% (DE = 5.5) in the 2 km radius, and 80.2% (DE = 19.3) in the 25 km radius. Among people declaring having received one dose, 71.3% and 71.0% had a card, respectively (Table 3). Based on the immunization cards, the at-least one-dose coverage decreased to 65.5% (DE = 18.9) and 56.4% (DE = 31.3), respectively. The at-least two-dose self-reported OCV coverage was 75.6% (DE = 17.3) in the 2 km radius and 54.2% (DE = 29.2) in the 25 km radius Among people declaring having received two doses, 64.9% and 76.9% had a card for the two doses, respectively (Table 3). Based on the immunization card, the at-least two-dose coverage decreased to 47.8% (DE = 20.7) and 41.6% (DE = 34.7), respectively. If we calculated the coverage among people having a card (N = 1885), we had 97.7% for one dose and 75.2% for two doses in 2 km radius; coverage in 25 km radius (N = 1663) were 98.9% and 73.9%, respectively.

**Table 3:** Overall vaccine coverage (by recall and card) in a 2 km (N = 2829) and 25 km (N = 2914) radius of Lake Chilwa, Malawi, December 2016.

				Oral Declaration		Card Presented
		N	n	% [95% CI]	n	% [95% CI]
2 km radius area						
	At least one dose	2829	2575	91.4 [88.9–93.9]	1837	65.5 [57.7–73.4]
	At least two doses	2829	2121	75.6 [68.8–82.4]	1376	47.8 [39.2–56.5]

25 km radius area						
	At least one dose	2914	2332	80.2 [73.6–86.7]	1644	56.4 [46.0–66.8]
	At least two doses	2914	1573	54.2 [44.2–64.3]	1210	41.6 [30.8–52.5]

A total of 74 individuals (2.6% [95% CI: 0–5.3]) reported having been vaccinated with more than two doses in the 2 km radius and 18 (0.6%, [95% CI: 0–1.4]) in the 25 km radius. In the 2 km radius, the second campaign increased the at-least one-dose vaccine coverage from 64.5% reached after the first campaign to 91.4%, and the at-least two-dose coverage from 52.4% to 75.6%.

In the 2 km radius, the at-least two-dose vaccine coverage was 72.0% in 1–4 years old, 80.2% in 5–14 years old and 73.8% in 15 years and older; in the 25 km radius, the coverages were 53.2%, 58.0% and 52.0%, respectively (Table 4). In individuals who reported living in floating homes or on islands at some point during the year, the self-reported at-least two-dose vaccine coverage was 92.2% in the 2 km radius (Table 4). Among them (n = 418), 35.1% (n = 147) took their second dose by DOV (n = 112 in February-March, n = 35 in November-December), 29.2% (n = 122) by self-delivery strategy (n = 39 in February-March, n = 80 in November-December and n = 3 unknown) and 35.6% (n = 149) by 2nd dose community supervised delivery strategy (n = 149 in February-March).

**Table 4:** Vaccine coverage assessed by recall per age groups and in individuals living on floating home or on islands at some point in the year, in a 2 km and 25 km radius of Lake Chilwa, Malawi, December 2016.

	Population in a 2 km radius of the lake			Population in a 25 km radius of the lake		
	N	n	% [95% CI]	N	n	% [95% CI]

1–4 years old						
At least one dose	518	470	90.8 [87.3–94.3]	575	462	80.7 [73.4–87.9]
At least two doses	518	373	72.0 [63.9–80.2]	575	305	53.2 [42.4–63.9]

5–14 years old						
At least one dose	901	852	94.7 [92.4–97.1]	968	839	86.7 [80.9–92.6]
At least two doses	901	722	80.2 [73.4–86.9]	968	560	58.0 [48.0–68.1]

≥15 years old						
At least one dose	1410	1253	89.6 [86.5–92.7]	1371	1031	75.4 [67.9–82.8]
At least two doses	1410	1026	72.8 [65.5–80.1]	1371	708	52.0 [41.7–62.2]

Individuals living on floating homes or islands at some point in the year						
At least one dose	457	443	97.1 [95.0–99.3]	239	220	91.7 [84.8–98.6]
At least two doses	457	418	92.2 [87.3–97.0]	239	201	83.7 [70.3–97.1]

The main reason for non-immunization was the absence during the campaigns, with 63.8% in the 2 km radius and 37.5% in the 25 km radius. The three main reasons for not having received the second dose included: vaccine shortage (31% and 32%, respectively), absence during the campaigns (27% and 26%, respectively), and absence during the first round (16% and 19%, respectively) (Table 5).

**Table 5:** Reasons for non-immunization with one or two doses among non or partially vaccinated individuals in a 2 km and 25 km radius of Lake Chilwa, Malawi, December 2016.

		Population in a 2 km radius		Population in a 25 km radius	
		n	%	n	%
Reasons for non-vaccination					
	Total of non-vaccinated individuals	254		582	
	Absent	162	63.8	218	37.5
	No vaccine available	17	5.7	104	17.9
	Not informed	14	5.5	127	21.8
	Not aware of eligibility for immunization	9	3.5	25	4.3
	Fear of AEFI^a^	3	1.2	8	1.4
	Bad taste or smell	3	1.2	0	NA
	Lack of confidence in vaccine	3	1.2	5	0.9
	Other reasons	36	14.2	79	13.6
	Not reported	7	2.8	7	1.2
	Place of immunization unknown	0	NA	5	0.9
	Long waiting time	0	NA	4	0.7

Reasons for having taken only one dose					
	Total with only one dose	454		759	
	No vaccine available	141	31.1	243	32.0
	Absent during the campaign	122	26.9	200	26.4
	Absent during 1st round	72	15.9	142	18.7
	Unaware that immunization needs two doses	15	3.3	80	10.5
	Place of immunization unknown	8	1.8	10	1.3
	Fear of AEFI	7	1.5	8	1.1
	Bad taste or smell	2	0.4	5	0.7
	Other reasons	75	16.5	48	6.3
	Not reported	12	2.6	15	2.0
	Long waiting time	0	NA	8	1.0

*NA*: Not applicable.

a Adverse events following immunization.

Among individuals who took their first dose in November-December 2016 and therefore received Euvichol, 6% and 3.4% reported presenting an adverse event in the 24 h following immunization, in the 2 km and 25 km radius, respectively. Nausea, vomiting, and diarrhea were the most frequently reported symptoms (Table 6).

**Table 6:** Adverse events occurred in the 24 h after immunization in individuals vaccinated with Euvichol® during the November-December OCV campaign in a 2 km (N = 718) and 25 km (N = 1548) radius of Lake Chilwa, Malawi, December 2016.

		Population living in 2 km radius		Population living in 25 km radius	
		N	%	N	%
Adverse events in the 24 h following immunization					
	Yes	43	5.0 [0.1–9.8]	52	3.4 [1.5–5.3]
	Yes, one	43	6.0	51	3.3
	Yes, two	0	NA	1	
	Not reported	2	0.3	0	NA

Symptoms					
	Nausea	39	90.7	36	69.2
	Diarrhea	2	4.7	8	15.4
	Vomiting	2	4.7	1	1.9
	Abdominal pain	0	NA	3	5.8
	Weakness	0	NA	2	3.9
	Headache	0	NA	1	1.9
	Fever	0	NA	1	1.9

After which dose					
	Dose 1	42	97.7	36	69.2
	Dose 2	1	2.3	15	28.9
	Not reported	0	NA	1	1.9

Health center attendance if adverse event					
	Yes	2	4.7	1	1.9
	Not reported	0	NA	6	11.5

*NA*: Not applicable.

## 4 Discussion

The one-dose vaccine coverage achieved after the two campaigns were quite high (94% for the 2 km radius area and 80% for the 25 km radius area). The immediate protection was expected to be high in the perspective of the 2017 cholera season. In the 2 km radius of the lake, three quarters of the population was estimated to be fully vaccinated. In this population, the opportunities of getting two vaccine doses was high since four vaccination rounds were organized from February to December. The coverage was also particularly high (92%) among those living on the lake at some point in the year. In the 25 km radius, half of people reported having received only one OCV dose. The individual protection may therefore last shorter. The highest coverage was reported in school-age children, while absence and vaccine shortage were the main reasons for non-immunization. The proportion of side effects in the 24 h following immunization was negligible.

Malawi has conducted several OCV campaigns since 2015. Following the reactive campaign conducted in 19 refugees camps (Nsanje district, 2015), the at-least-one-dose coverage was 86% and the two-dose coverage of 68% [5]. Following the pre-emptive campaign conducted in Kapise refugee camp (Mwanza district, March-April 2016), the coverage was 93% and 56%, respectively [6]. The one-dose vaccine coverage measured in our survey was therefore similar while our at least two-dose coverage measured was higher in the 2 km radius area of Lake Chilwa. However, it was the first time that some mass campaigns were organized in general population. Considering the periodicity of cholera outbreaks, the impact of this coverage on future epidemic occurrence remain also unknown.

After the two successive campaigns, the one-dose vaccine coverage estimated in our survey provides an immediate individual protection if an outbreak starts and should limit the spread of the outbreak [7], [8], [9]. At the time of our survey, however, there was no ongoing outbreak. In February-March, more than half of individuals living within the 2 km radius of the lake had been vaccinated with only one dose. According to a recent study conducted in Bangladesh, the single-dose protection at 6 months may not differ significantly from protection at one year obtained after two doses, suggesting a similar short-term protection [9], [10]. Individuals immunized during the first campaign were potentially still protected at survey time. However, in the absence of a second dose in the following months or a booster effect due to natural exposure to *Vibrio cholerae* as in endemic settings, we do not have evidence of longer protection.

Two doses of killed whole-cell vaccines provide a high level of direct protection probably lasting at least three years as well as some herd protection [11], [12], [13], [14], [15]. The current population in the 2 km radius should therefore be adequately protected for at least the three next cholera seasons since the large majority of fully vaccinated individuals had received their two doses during the same campaign respecting the two weeks interval between doses. A study conducted in Bangladesh estimated that 50% of OCV coverage with Shanchol® could control cholera transmission in endemic settings where people have natural immunity [16]. In our context, less endemic than Bangladesh, we do not know what is the degree of natural immunity of the population and therefore if the 54% vaccine coverage reached in the 25 km radius will be enough to control cholera transmission. This would require conducting prospective follow-up.

According to our findings, the observed low two-dose coverage is partly due to the vaccine shortage, observed in some areas. The number of vaccines requested from the Global Task Force on Cholera Control was based on an estimate of 90,000 people living in a 25 km perimeter of the lake. In December 2016, the population was 2.4-fold higher than previously estimated based on a local census. In addition, the immunization points were open only one day per week in the targeted villages, which limited vaccination opportunities. Consequently, absence during the campaign and vaccine unavailability were often reported as the main reasons for incomplete or non-immunization. Furthermore, the catchment area of each immunization point included several villages, and the distance to the vaccination point may have been a significant obstacle. Such a strategy may explain the high coverage variability between clusters that we measured in our survey, illustrated by a high observed design effect.

In the population living in the 25 km radius, vaccine coverage was higher among school children. As many young women attend the health centers for the ante- and post-natal follow-up, they may have been properly sensitized to the cholera campaign at this time. The medical staff have probably encouraged them to have all their children immunized. According to previous studies, the population the most affected by cholera in this area is the fishermen, and more specifically the individuals living on *Zimboweras* and islands at some point of the year [2], [3]. More than half of these individuals received their second dose through simplified delivery strategies, self- and community-supervised delivery. Our results showed therefore the high added value of such strategies to reach an acceptable coverage in this hard-to-reach population. As other fishermen may come from outside the surveyed districts, including from the Mozambican districts which border the lake, the level of herd immunity in this at-risk population remain unknown.

Because Euvichol® has been only prequalified in December 2016, few data exist on AEFI occurrence. We found the vaccine is well tolerated. No severe adverse events were reported in the 24 h following vaccination. The symptoms recorded were similar to those reported for Shanchol® or Dukoral® vaccines [17], [18], [19].

Our study has several limitations. First, the long time span between the first reactive campaign (February-March) and the vaccine coverage survey (December) have resulted the loss of a significant number of immunization cards; and the occurrence of recall bias regarding dates and immunization places could not be excluded. This means that the vaccine coverage estimates based on oral declarations only might have overestimated the true figure. Second, if a family member was absent at the time of the interview, the representative replying on their behalf may be unaware if they were vaccinated during the first campaign, leading to underreporting.

The November-December 2016 campaign targeted everyone older than one year in a 25 km radius. This means that the majority of individuals vaccinated during this campaign were not the most at-risk population that is fishermen living in *Zimboweras*. Even if this population was fully vaccinated, we have no certainty to control the next epidemic if a large number of susceptible fishermen were to arrive on the lake from outside the districts. Such immunization strategies based on two successive campaigns present a high logistical cost regarding materials, vehicles, and human resources deployment, and the overall protection achieved remain unknown if fishermen are the target audience. Regular catch-up campaigns or another strategy specifically targeting fishermen may be envisaged for the future. A potential novel approach might be a systematic immunization of new fishermen arriving on the lake in well identified immunization points. The active involvement of the fishermen communities is indispensable and adequate communications need to be developed.

## 5 Conclusion

In conclusion, two OCV campaigns conducted at an nine-month interval targeting everyone older than one year in areas hosting mobile fishermen have offered an important opportunity to achieve a high vaccine coverage of the most at-risk population. However, knowing that the fishermen population changes over time, we do not know if such vaccine coverage will be sufficient to control an outbreak if additional fishermen arrive from other districts. Innovative strategies specifically targeting fishermen need to be assessed to control epidemics in this well-defined, at-risk population. Proposing an efficient strategy is a priority since this type of mobile population with limited access to Water - Sanitation and Hygiene (WASH) is at-risk of cholera in many other settings.
